# Xiyanping injection combined with acitretin for psoriasis vulgaris: A systematic review and meta-analysis

**DOI:** 10.3389/fphar.2022.971715

**Published:** 2022-09-06

**Authors:** Man-Ning Wu, Li-Jia-Ming Zhou, Dong-Mei Zhou

**Affiliations:** Department of Dermatology, Beijing Hospital of Traditional Chinese Medicine, Capital Medical University, Beijing, China

**Keywords:** Xiyanping injection, traditional Chinese medicine, psoriasis, acitretin, meta-analysis

## Abstract

**Background:** Psoriasis represents the chronic, recurrent and inflammatory disorder. The Traditional Chinese Medicine Xiyanping injection (XYP) is extensively applied in China for treating diverse inflammatory disorders, such as bronchitis, viral pneumonia or upper respiratory tract infection. XYP may offer a potential treatment for psoriasis vulgaris (PV). This study focused on analyzing whether XYP combined with acitretin was effective and safe.

**Methods:** The present meta-analysis was carried out in line with guidelines of Preferred Reporting Items for Systematic Reviews and Meta-Analyses (PRISMA). This systematic review was registered in PROSPERO (CRD42022333273). Besides, relevant randomized controlled trials (RCTs) that compared XYP plus acitretin with acitretin alone for treating PV were searched from several databases from their inception till May 2022. In addition, this work utilized RevMan5.4 to conduct risk assessment as well as meta-analysis.

**Results:** This meta-analysis selected altogether 10 RCTs including 815 subjects. Upon quality assessment, the RCTs mainly had low or unclear risk. According to our meta-analysis results, relative to acitretin monotherapy, XYP plus acitretin increased the total clinical effective rate, as evidenced by Psoriasis area and severity index score (PASI)-20, PASI-30 and PASI-60 in patients with PV [risk ratio (RR) = 1.23 Z = 4.87, *p* < 0.00001, 95% confidence interval (CI): 1.13–1.34; RR = 1.29, Z = 3.89, *p* = 0.009, 95% CI: 1.07 to 1.55; and RR = 1.31, Z = 3.89, *p* = 0.0001, 95% CI: 1.14–1.49]; the reduced levels of TNF-α, MCP-1 and RANTES, the alleviated side effects resulting from acitretin like itchiness (RR = 0.54, 95% CI: 0.4 to 0.74, *Z* = 3.94, *p* < 0.0001), and the increased levels of aminotransferases and dyslipidemia (RR = 0.5, 95%CI = 0.29, 0.86, *p* = 0.01; and RR = 0.41, 95% CI = 0.23, 0.75, *p* = 0.004).

**Conclusion:** As suggested in the present meta-analysis, XYP combined with acitretin effectively and safely treats PV.

**Systematic Review Registration:** https://www.crd.york.ac.uk/prospero/display_record.php?ID=CRD42022333273, identifier PROSPERO 2022 CRD42022333273.

## Introduction

As one of the inflammatory, autoimmune disorders, psoriasis mainly manifests in the joints and skin and affects 1–3% of global population (Papp et al., 2013). Psoriasis vulgaris (PV) represents the frequently seen clinical phenotype of psoriasis. Typically, the disease presents with well-defined erythematous plaques under the coverage of silvery-white scales. It affects the elbows, knees, trunk, and scalp in a symmetrical pattern (Korman, 2020). Additionally, it also has a significantly negative impact on the physical, emotional, and psychosocial well-being of the patients (Langley et al., 2005). In the Traditional Chinese Medicine theory, dermatological conditions are treated for centuries, which is safe and effective for the treatment of psoriasis (May et al., 2012). Traditional Chinese Medicine injection is the preparation that shows distinct Chinese features (Li et al., 2018). For centuries, Traditional Chinese Medicine injection is adopted to treat, prevent and cure diseases (Liu et al., 2015). The Traditional Chinese Medicine injection Xiyanping (XYP) is made from andrographolide, a compound found in the well-known medicine Andrographis paniculata (Burm. f.) Nees that can be used for dissipating blood stasis and clearing heat toxicity. It possesses strong activities of antivirus and anti-bacterium, and high safety without any significant resistance to antibiotics, therefore, it is extensively utilized to treat infectious disorders like bacillary dysentery, tonsillitis, and bronchitis in China (Jia et al., 2018; Yang et al., 2019). XYP is identified to be the option with the highest effectiveness to replace antibiotics (Yang et al., 2019). In addition, it was reported that the adverse events of using XYP were mainly allergic reactions, involving diseases in respiratory system, integumentary system, digestive system, and so on. Adverse drug reactions happened mostly when applying XYP within 30 min (70.2%) and the majority (95.7%) were cured when treated in time. (Chen et al., 2018). Thus, it is significant to inquire the detailed allergic history from the patients and consider the allergic constitution before taking XYP. As reported in some recent studies, XYP combined with acitretin can be applied clinically to treat PV. In studies, XYP is used in combination with acitretin to reduce the Psoriasis Area and Severity Index (PASI) score, in psoriatic cases, improve levels of chemokines (MCP-1, RANTES) and inflammatory factors (TNF-α), and decrease the incidence of adverse reactions (Zhao, 2011; Bao, 2016; Jin, 2016; Ti, 2016; Zhen, 2017; Hong, 2018; Hui, 2018; Si, 2018; Zi, 2018; Hong, 2020). Nonetheless, relevant studies are still lacking. For instance, no study has summarized those aforementioned studies or evaluated the evidence level. Consequently, it is necessary to systemically review studies concerning whether XYP combined with acitretin is more effective and safer than acitretin alone for the treatment of PV.

## Materials and methods

The present meta-analysis was carried out in line with guidelines of Preferred Reporting Items for Systematic Reviews and Meta-analyses (PRISMA).

### Study screening criteria

This work selected RCTs in line with PICOS criteria: 1) Population: the study subjects were Psoriasis vulgaris cases, with no restriction on age, sex, or disease severity or stage. For PV, there is no limit to first diagnosis or recurrence. 2) Intervention: XYP injection plus acitretin should be selected as the experimental intervention 3) Comparison: acitretin alone should be the control intervention. Both groups were allowed to receive other treatments, such as topical ointment, but such treatments should be consistent between the two groups. 4) Outcomes: the primary outcome was total effective rate and PASI score, and the secondary outcomes were the level of inflammatory and chemotactic factors, side effects, dermatology life quality index, visual analog scale score, etc. 5) Study design: RCTs only published in English or Chinese with no restriction on year of publication or country, and only human RCT studies can be included in the review, while other kinds of study will be excluded. 6) Exclusion criteria: such as observational studies, retrospective analyses, self-controlled trials, case reports, reviews, patient series, animal experiments, etc.

### Literature search strategy

**FIGURE 1:**
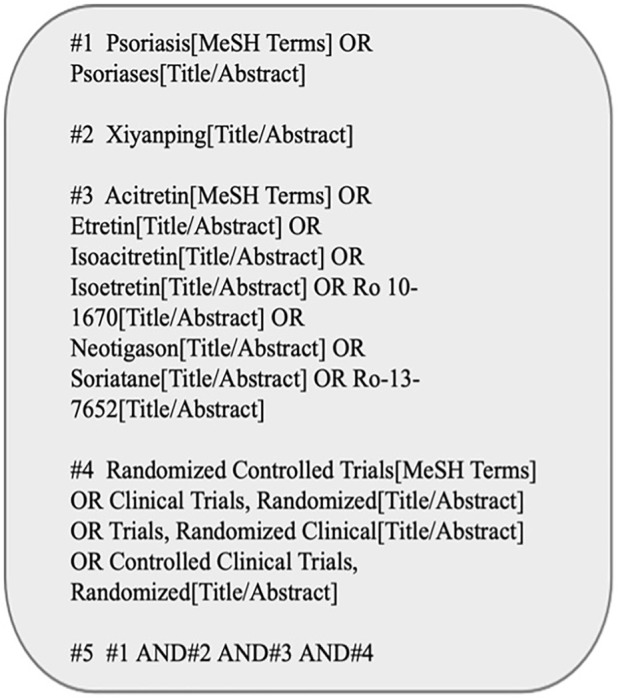
The search strategy of PubMed.

The English databases (namely, Web of Science, The Cochrane Library, Embase, PubMed) together with Chinese databases (Wan fang Database, China National Knowledge Infrastructure (CNKI), Sino Med, Baidu Scholar, VIP Database for Chinese Technical Periodicals) were systemically searched from their inception till May 2022. Figure 1 presents the PubMed database search strategy as an example.

### Data collection and bias risk evaluation

Two reviewers (MNW, LJMZ) independently selected RCTs and collected data, and all results were cross-checked. Any disagreement between the two researchers was solved by mutual negotiation or the third party (DMZ). Data were extracted in the table, including first author, publication year, country, sample size, interventions, as well as outcomes. In addition, the Cochrane Collaboration’s risk of bias tool was adopted for assessment, which mostly contained several aspects shown below, 1) generation of random sequence; 2) concealment of allocation; 3) method of blinding; 4) sufficiency of outcome information; 5) the presence/absence of a selective report; and 6) additional biases. Two reviewers independently assessed study quality. Any disagreements were solved through discussion with a third reviewer (DMZ).

### Statistical analysis

All statistical analyses were completed using Review Manager 5.4 software. Continuous variables were displayed as mean difference (MD), whereas dichotomous variables as risk ratio (RR) and 95% confidence intervals (CI). The potential heterogeneity was detected by χ2 test between studies. The fixed-effects model (FEM) was adopted when there was no heterogeneity (*p* > 0.1, I^2^<50%); or else (I^2^ ≥ 50%), the random-effects model (REM) was adopted. In the case of distinct heterogeneity (I^2^ ≥ 75%), sensitivity analysis was performed.

## Results

### Study search results

**FIGURE 2:**
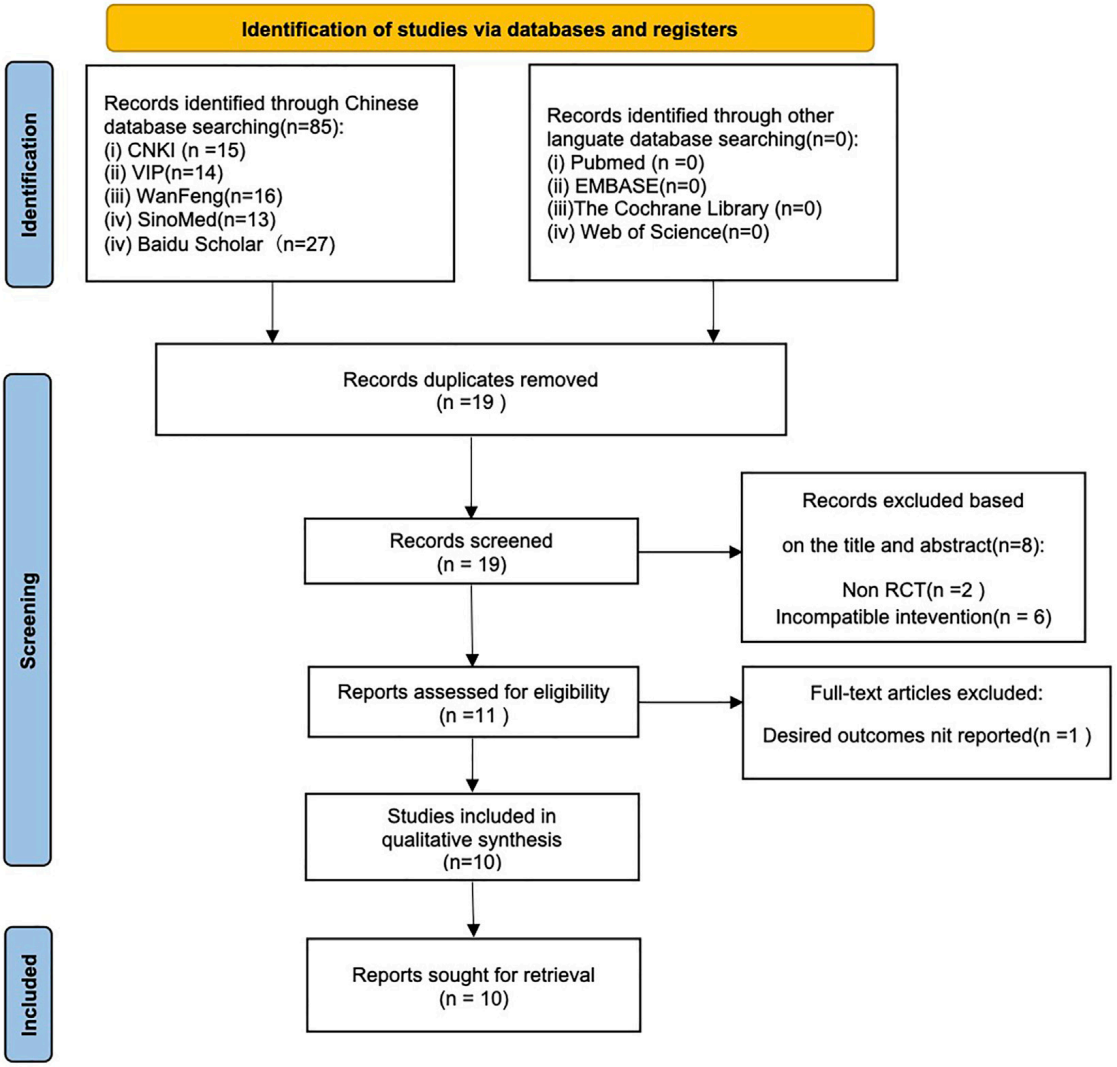
Flowchart showing the study screening procedure.

Altogether 85 records were identified by searching the databases or additional sources. There were 19 studies identified by preliminary search following our preset search strategy. Eight duplicate articles were eliminated after careful title and abstract reading. Besides, another one study was eliminated based on our inclusion and exclusion criteria, eventually, 10 studies were selected (Zhao, 2011; Bao, 2016; Jin, 2016; Ti, 2016; Zhen, 2017; Hong, 2018; Hui, 2018; Si, 2018; Zi, 2018; Hong, 2020) (Figure 2).

### Characteristics of the selected studies

This meta-analysis included 10 RCTs involving altogether 819 participants (*n* = 447 and *n* = 372 from the treatment and control groups, separately). There were 26–73 and 22–73 individuals participating in the treatment and control groups, separately. Meanwhile, the age of patients in treatment group ranged from 16 to 77 years, while that in control group ranged from 18 to 79 years. In addition, the participants in nine studies were recruited from hospitals, while those in one study were from a health center. Only one RCT included participants diagnosed with progressive PV, whereas the remaining studies did not describe the stage of psoriasis patients. In 3 studies, topical ointment was further applied to patients both in intervention and control groups. Table 1 displays more details of study descriptions.

**TABLE 1:** Characterization of selected studies.

Author, year, country	Sample size (T/C)	Age		Baseline PASI score		Intervention		Treatment period	Outcome indicators	
		T	C	T	C	T	C		Total effective rate	Other indicators
WU, 2016, China	72 (36/36)	19–71				XYP 10 ml ivgtt q.d.+ Oral acitretin 0.5 mg/kg·d; compound flumetasone ointment	Oral acitretin 0.5 mg/kg·d; compound flumetasone ointment	1 month	Based on PASI 60	4)
Tang, 2018, China	96 (48/48)	58.6 ± 2.1	58.7 ± 2.3	10.15 ± 2.17	10.13 ± 2.18	XYP 250–500 mg ivgtt q.d. + Oral acitretin 10 mg t.i.d	Oral acitretin 10 mg t.i.d	30 days	Based on PASI 30	1)
		53–72	54–71							
Li, 2018, China	79 (40/39)	43.22 ± 10.53	44.19 ± 9.36	19.71 ± 3.79	18.28 ± 4.34	XYP 300 mg ivgtt q.d.+ Oral acitretin 10mg t.i.d	Oral acitretin 10 mg t.i.d	14 days	Based on PASI 20	1) 5)
		20–71	21–72							
Yang, 2018, China	146 (73/73)	40.4 ± 5.1	40.3 ± 5.2	-	-	XYP 300 mg ivgtt q.d.+ Oral acitretin 10–20 mg t.i.d	Oral acitretin 10–20 mg t.i.d	Oral medicine:4 weeks	Based on PASI 20	2) 4)
		20–60	21–62					Injection:2 weeks		
Duan, 2017, China	84 (42/42)	40.08 ± 6.39	40.11 ± 6.43	17.62 ± 3.71	16.19 ± 3.14	XYP 300 mg ivgtt q.d. + Oral acitretin 20 mg t.i.d	Oral acitretin 20 mg t.i.d	Oral medicine:1 month	Based on PASI 20	1) 2) 3) 4)
		20–64	19–62					Injection:2 weeks		
Wang, 2020, China	78 (39/39)	57.35 ± 2.6	58.35 ± 2.5	13.43 ± 2.36	12.68 ± 2.03	XYP 250 mg ivgtt q.d. + Oral acitretin 10 mg t.i.d; compound flumetasone ointment,b.i.d	Oral acitretin 10 mg t.i.d; compound flumetasone ointment,b.i.d	30 days	Based on PASI 60	1) 4)
		19–77	20–79							
Jia, 2016, China	100 (50/50)	18–61		18.7 ± 3.8	17.2 ± 3.2	XYP 250 mg ivgtt q.d. + Oral acitretin 10–20 mg t.i.d	Oral acitretin 10–20 mg t.i.d	Oral medicine:4 weeks	Based on PASI 20	1) 3) 4)
								Injection:2 weeks		
Zhao, 2018, China	120 (60/60)	43.2 ± 11.5	45.1 ± 12.3	18.72 ± 3.89	18.31 ± 3.65	XYP 300 mg ivgtt q.d. + Oral acitretin 10 mg t.i.d	Oral acitretin 10 mg t.i.d	Oral medicine:4 weeks	Based on PASI 20	1) 2) 3) 4)
		19–72	21–75					Injection:2 weeks		
Zhao,201, China	48 (26/22)	16∼67	17∼70	-	-	XYP 10 ml ivgtt q.d. + Oral acitretin 0.5 mg/kg·d	Oral acitretin 0.5 mg/kg·d	1 month	Based on PASI 60	4)
Chen, 2016, China	66 (33/33)	18–68	17–70	13.44 ± 2.37	12.69 ± 2.04	XYP 250 mg ivgtt q.d. + Oral acitretin 10 mg t.i.d; compound flumetasone ointment,b.i.d	Oral acitretin 10 mg t.i.d; compound flumetasone ointment,b.i.d	1 month	Based on PASI 60	1) 4)

Note: XYP, Xiyanping injection; T: treatment group; C: control group; ivgtt, intravenously guttae; q.d., once daily; bid, twice daily; t.i.d, thrice daily; PASI, psoriasis area and severity index; 1)PASI, score; 2) RANTES, and MCP-1levels; 3) TNF-α, level; 4) side effect; 5) recurrence rate.

### Bias risk of the selected RCTs

#### Sequence generation and allocation concealment

Li (Si, 2018) and Wang (Hong, 2020) were the only two studies that used the random number table, which were therefore identified with low bias risk. The remaining studies simply mentioned “random” but did not describe the method in detail. As a result, they were classified as unclear risk of bias. Since all the studies did not mention whether they accessed the allocation concealment, they were considered with unclear bias risk.

#### Blinding, insufficient outcome information and selective reporting

All the articles did not state whether blinding was utilized and were thus considered with unclear bias risk. With regard to insufficient outcome information, all RCTs were considered to have low risk, since no dropout was reported. In terms of selecting reporting, the selected RCTs were considered to have low risk.

#### Other potential biases

**FIGURE 3:**
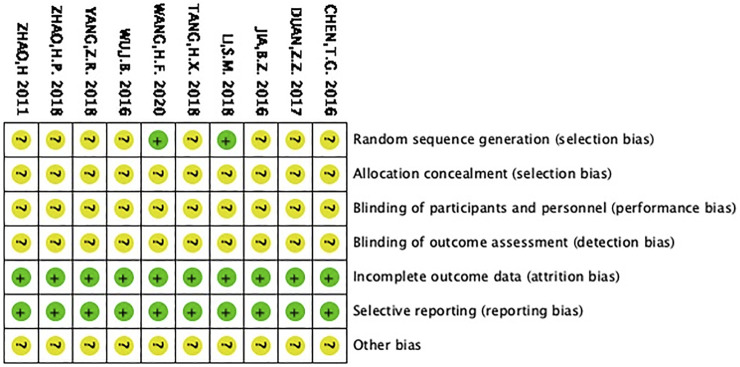
Graph showing the risk of selection bias. (+) low risk, (?) unclear risk.

All the RCTs did not mention other potential biases, which were thus accessed to have unclear bias, as shown in Figure 3.

### Meta-analysis on total effective rate

The selected studies suggested that XYP plus acitretin has better total effective rate after treatment. Total effective rate = the number of cases of cure + effective (+improvement)/total number of cases × 100%. Efficacy index = PASI total score before treatment − PASI total score after treatment/PASI before treatment; cure: curative efficacy index >95%; effective: curative efficacy index 60–95%; improved: curative efficacy index 20/30∼60%; and ineffective: curative efficacy index <20/30%. For example, PASI60 means the number of cases achieved a 60% reduction in PASI score.

There were five RCTs (Bao, 2016; Zhen, 2017; Hui, 2018; Si, 2018; Zi, 2018) using PASI20 as the criterion, 1 (Hong, 2018) adopting PASI30 as the criterion, and 4 (Zhao, 2011; Jin, 2016; Ti, 2016; Hong, 2020) applying PASI60 as the criterion. All the RCTs were combined as three groups, as shown below.

**FIGURE 4:**
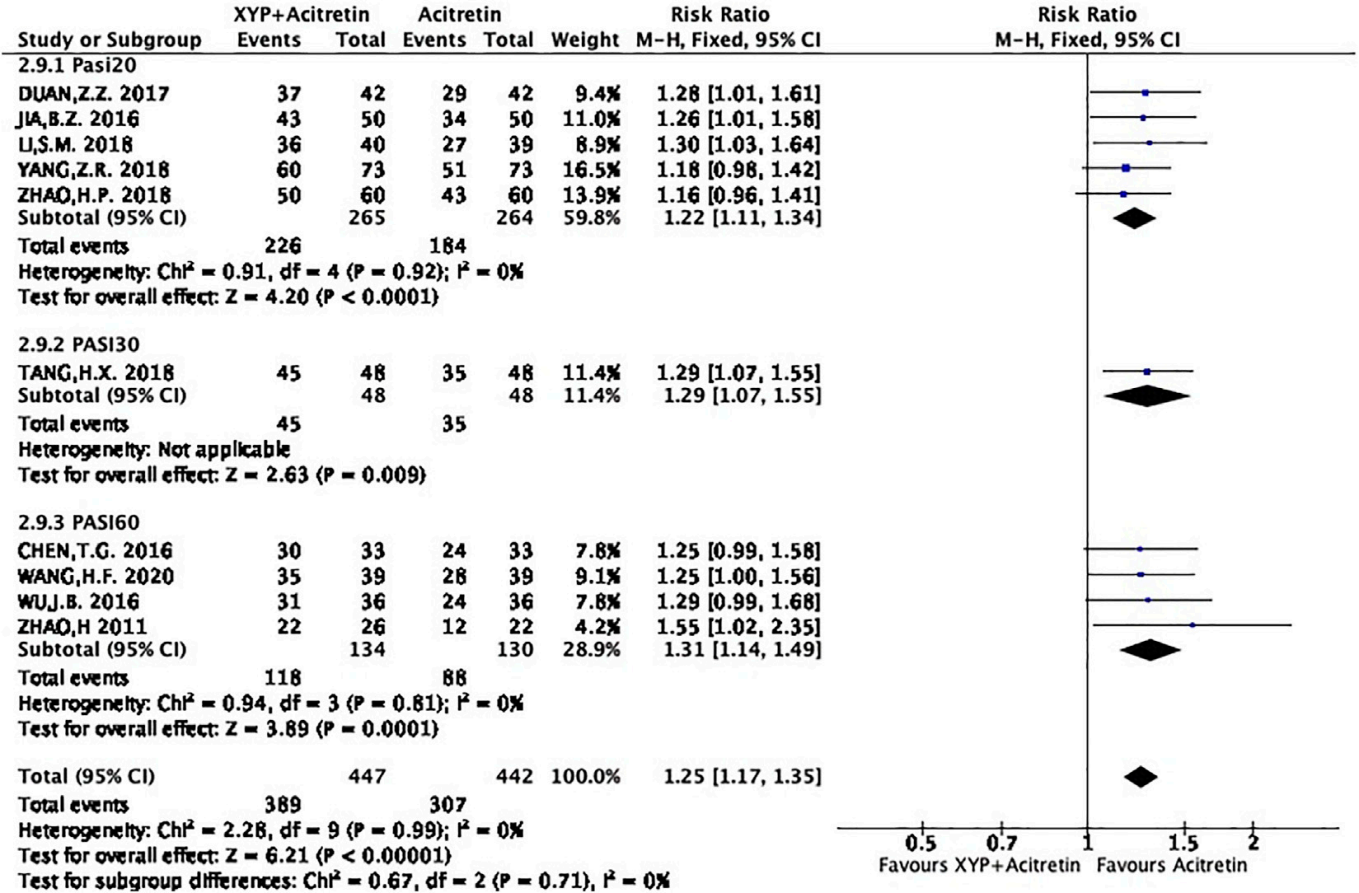
Forest plots showing RR for Total effective rate.

Group1: PASI20: 5 RCTs mentioned 410 subjects (50.3%) achieving the 20% reduced PASI. On the whole, there was no heterogeneity among different studies (*p* = 0.92, I^2^ = 0%), so a FEM was used. The results are displayed in Figure 4. In the meta-analysis, it was found that treatment group achieved an increased total effective rate compared with control group, with significant difference (*n* = 5, RR = 1.22 Z = 4.2, *p* < 0.0001, 95% CI: 1.11 to 1.34).
Group2: PASI30: There were 80 participants (9.8%) who achieved a 30% reduction in PASI. As shown in Figure 4, patients in combination group showed a significantly higher total effective rate than those in acitretin group (n = 1, RR = 1.29, Z = 3.89, *p* = 0.009, 95% CI: 1.07 to 1.55).
Group:2: PASI60: There were 206 participants (25.2%) achieving a 60% reduction in PASI. No distinct heterogeneity was detected among different studies (*p* = 0.81, I^2^ = 0%), so this work used a FEM. According to Figure 4, participants receiving XYP combined with acitretin had a markedly increased total effective rate compared with patients receiving acitretin alone [*n* = 4, RR = 1.31, *Z* = 3.89, *p* = 0.0001, 95% CI: 1.14 to 1.49].

### Meta-analysis on the PASI score

A patient’s PASI is a measure of overall psoriasis severity and coverage. It is a commonly-used measure in clinical trials for psoriasis treatments. PASI consists of two major steps: 1) Calculating the body surface area covered with lesions and assessment of the severity of lesions. 2) In turn consists of assessing lesions’ erythema, induration and scaling. All calculations are combined into a single score (PASI Score) in the range of 0 (no psoriasis on the body) and up to 72 (the most severe case of psoriasis).

**FIGURE 5:**
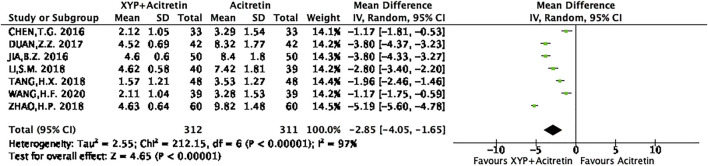
Forest plots showing the PASI score.

**FIGURE 6:**
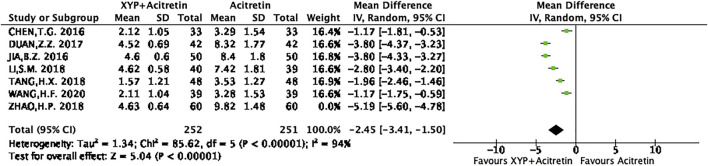
Forest plots showing the sensitivity analysis of PASI score.

There were seven RCTs (Bao, 2016; Zhen, 2017; Hong, 2018; Hui, 2018; Si, 2018; Hong, 2020) reporting the actual PASI score. Altogether of 312 subjects were selected into intervention group, while 311 were included into control group. Differences in data from these seven studies were significant, as revealed by heterogeneity test (*p* < 0.00001, I^2^ = 97%), so a REM was used. The results are presented in Figure 5. Based on the above findings, treatment group had decreased PASI scores compared with control group, and the difference was statistically significant (*n* = 7, MD = -2.85, 95% CI: −4.05 to −1.65, Z = 4.65, *p* < 0.00001). This work also conducted sensitivity analysis, which revealed no distinct alterations of the overall effect when one specific article was excluded, indicating that our meta-analysis results were stable. The result of sensitivity analysis was shown in Figure 6.

### Meta-analysis on side effects

#### Dry mouth

**FIGURE 7:**
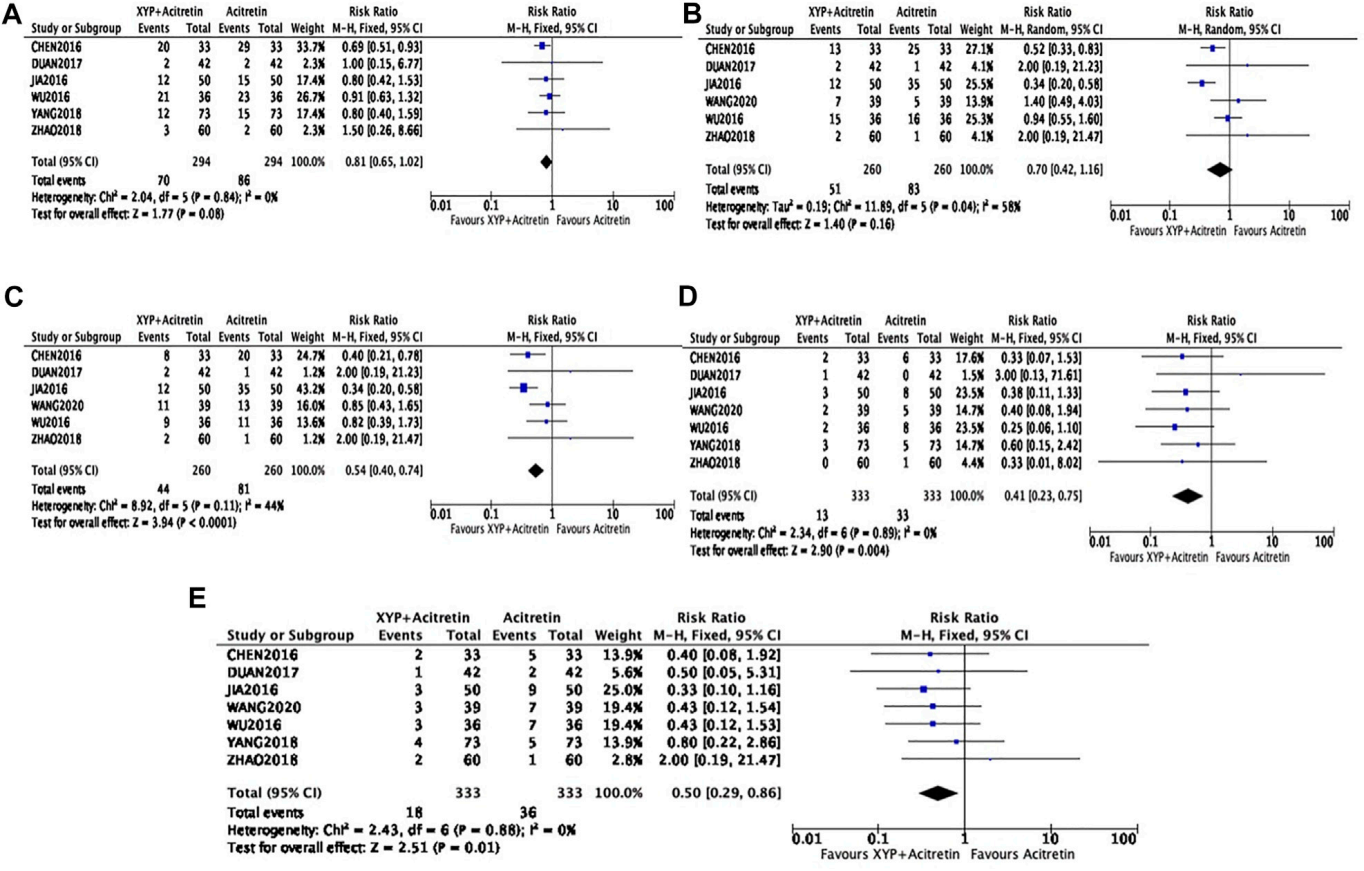
Forest plots showing side effects. Dry mouth: **(A)**; Dry skin: **(B)**; Itchiness: **(C)**; Dyslipidemia: **(D)**; Elevation of aminotransferases: **(E)**.

The incidence of dry mouth after treatment was reported in six articles (Bao, 2016; Jin, 2016; Ti, 2016; Zhen, 2017; Hui, 2018; Zi, 2018). There were altogether 588 cases involved, including 294 in intervention group and 294 in control group. Heterogeneity between studies was not detected (*p* = 0.84, I^2^ = 0%). According to Figure 7A, our FEM-based meta-analysis suggested that the dry mouth rate was not significantly different between the two groups (*n* = 6, RR = 0.81, 95% CI: 0.65 to 1.2, Z = 1.77, *p* = 0.08).

#### Dry skin and itchiness

There were six RCTs (Bao, 2016; Jin, 2016; Ti, 2016; Zhen, 2017; Hui, 2018; Hong, 2020) involving a total of 520 participants reporting the incidence of dry skin. There was a modest heterogeneity in statistics (*p* = 0.04, I^2^ = 58%), therefore, the REM was used, as shown in Figure 7B. In the meta-analysis, the incidence of dry skin was not statistically different between the experimental and control groups (*n* = 6, RR = 0.7, 95% CI: 0.42 to 1.16, Z = 1.4, *p* = 0.16). Additionally, six RCTs involving 520 cases (*n* = 260 in experimental and control groups, separately) investigated the incidence of itchiness. There was a low heterogeneity in statistics (*p* = 0.11, I2 = 44%), so the FEM was used. According to Figure 7C, our meta-analysis findings revealed decreased incidence of itchiness in experimental group compared with control group, with significant difference (*n* = 6, RR = 0.54, 95% CI: 0.4 to 0.74, *Z* = 3.94, *p* < 0.0001).

#### Dyslipidemia and elevation of aminotransferases

Seven studies (Bao, 2016; Jin, 2016; Ti, 2016; Zhen, 2017; Hui, 2018; Zi, 2018; Hong, 2020) recruiting a total of 666 participants reported the significantly decreased incidence of dyslipidemia and elevation of aminotransferases (*n* = 7, RR = 0.41, 95% CI = 0.23, 0.75, *p* = 0.004; *n* = 7, RR = 0.5, 95% CI = 0.29, 0.86, *p* = 0.01), with no heterogeneity being detected (*p* = 0.89, I^2^ = 0; *p* = 0.88, I^2^ = 0), as shown in Figures 7D,E.

The selected RCTs did not report any severe side effect.

### Levels of TNF-α, MCP-1, and RANTES

**FIGURE 8:**
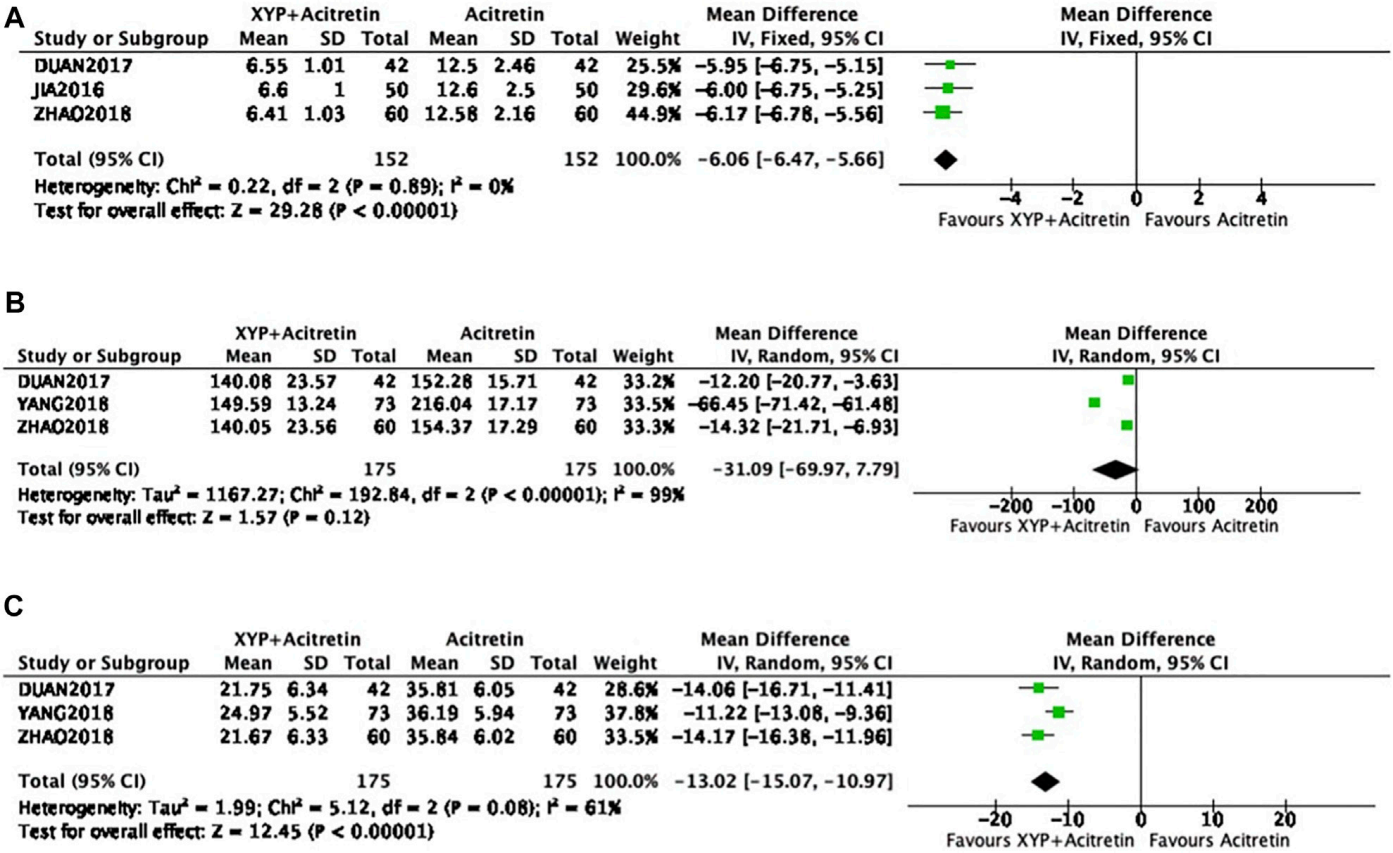
Forest plots showing TNF-α, MCP-1, and RANTES levels. TNF-α: **(A)**; MCP-1: **(B)**; RANTES: **(C)**.

There were three papers (Bao, 2016; Zhen, 2017; Hui, 2018) mentioning TNF-α level, including 152 from intervention and control groups separately. Since no heterogeneity was detected in statistics (*p* = 0.89, I^2^ = 0%), the FEM was used, as displayed in Figure 8A. The result of meta-analysis revealed that treatment group had decreased TNF-α level compared with control group, with significant difference (*n* = 3, MD = -6.06, 95% CI: −6.47 to −5.66, Z = 29.28, *p* < 0.00001). Additionally, three articles (Zhen, 2017; Hui, 2018; Zi, 2018) involving altogether 350 participants reported MCP-1 and RANTES levels. Modest to significant differences were detected (*p* < 0.00001, I^2^ = 99%; *p* < 0.08, I^2^ = 61%), so the REM was used (Figures 8B,C). According to our meta-analysis results, intervention group had decreased MCP-1 and RANTES levels compared with control groups, with significant differences [*n* = 3, MD = -6.06, 95% CI: −6.47 to −5.66, Z = 29.28, *p* < 0.00001; *n* = 3, MD = −12.8, 95% CI: −14.06 to −11.55, Z = 29.28, *p* < 0.00001].

As revealed by meta-analysis of MCP-1, the high heterogeneity and the possible publication bias were detected, so sensitivity analysis was conducted by removing the study by Yang (Zi, 2018). The result of the adjusted meta-analysis showed a significant difference (*n* = 2, MD = -13,41, 95% CI: −19.01 to −7,82, Z = 4.7, *p* < 0.00001), and there was no heterogeneity detected (*p* = 0.71, I2 = 0%).

## Discussion

Psoriasis accounts for a kind of inflammatory cutaneous disorder related to numerous medical conditions, which is reported in more than 60 million child and adult populations globally (Griffiths et al., 2021). Of all those psoriasis types, psoriasis vulgaris (PV) is the most common (Griffiths and Barker, 2007). Acitretin, a retinoid used to treat PV, has minor to severe side effects when taken over a long period of time (Nast et al., 2012). XYP injection, one of the injectable Traditional Chinese Medicine preparation, has heat clearing and detoxication effects. Recently, several clinical trials show that XYP is effective on treating PV, which decreases the side effect rate associated with acitretin. However, there is few evidence-based article providing proofs. This review analyzed the results of RCTs for investigating whether XYP plus acitretin was effective and safe for PV.

According to our meta-analysis results, XYP plus acitretin was effective on PV with regard to skin lesion (total effective rate, PASI score), TNF-α, MCP-1, RANTES levels, and the incidence of side effects. Firstly, XYP combined with acitretin achieved an increased total effective rate for treating PV, irrespective of disease severity and course. The combined treatment with XYP and acitretin significantly lowered the PASI score, a clinically key measure of PV. Secondly, based on our meta-analysis, XYP plus acitretin regulated the levels of inflammatory cytokines and chemokines closely related to PV pathogenesis. A growing number of studies demonstrate that psoriatic patients express high levels of TNF-α, MCP-1 and RANTES, all of which have critical effects on the PV pathogenesis. Meanwhile, they are strongly related to the PV severity (Raychaudhuri et al., 1999; Giustizieri et al., 2001; Lembo et al., 2014). In this meta-analysis, XYP combined with acitretin remarkably reduced the levels of TNF-α, MCP-1 and RANTES, implying that XYP possessed immune regulation and anti-inflammatory effects. Thirdly, with regard to side effects associated with acitretin, skin dry, itchiness, acute hepatocyte damage and hyperlipidemia are the well-known adverse events of acitretin (Katz et al., 1999). In our meta-analysis, XYP decreased the incidence of itchiness, hyperlipidemia, and the elevation of aminotransferase that was associated with liver injury (Katz et al., 1999). XYP can reduce the side effect rate of acitretin, making it easier for patients to receive treatment. There were high heterogeneities in PASI score and MCP-1 level upon further analysis. There are two possible reasons causing significant heterogeneity in PASI score. 1) It was judged by different researchers, which might be associated with deviation caused by human factors. 2) The baseline PASI scores of patients in different studies were not exactly the same, thus the PASI scores after treatment were apparently different between these studies. As for MCP-1, its mean level after treatment in control group of Yang’s study (Zi, 2018) remarkably increased compared with the remaining two control groups, which might cause a high heterogeneity.

For comprehensive result assessment and interpretation, this work explored the pharmacological mechanism. With regard to the effect on the improvement of skin lesion, andrographolide (Andro), the component of XYP, alleviated imiquimod-mediated psoriasis within mice that had reduced skin IL-1β and IL-23 levels, as revealed by Fenli Shao et al. (Shao et al., 2016). As for the regulation of TNF-α, MCP-1 and RANTES levels, Andro changed macrophage polarized status from pro-inflammatory state into anti-inflammatory state, reduced TNF-α and MCP-1 levels, and attenuated the up-regulation of RANTES after IL-1β stimulation (Wong et al., 2014; Shao et al., 2016; He et al., 2020). With regard to the decreased incidence of hyperlipidemia and the elevation of aminotransferase, Song Y et al. reported that Andro improved serum aminotransferase (ALT,AST), liver function, and lipid accumulation (TC,TG) through down-regulating TNF-α and NF-kB (Song et al., 2020). Lin L et al. also demonstrated that Andro treatment reduced the ALT and AST levels in mice with liver fibrosis, which was related to suppressing TLR4/NF-kB p50 and TGF-1/Smad2 pathways (Lin et al., 2018). In addition, the results obtained by Islam et al. demonstrated that Andro significant reduced TC and TG in rats with Porphyromonas gingivalis-induced hyperlipidemia (Islam, 2017).

XYP injection is associated with certain side effects as well, including itching, rash, chills, fever, irritability, breath shortness, palpitations, cyanosis or convulsions (Shi et al., 2019). In 2018, one meta-analysis including 1,578 articles was conducted to analyze side effect distribution features. The results reported a 1.8% adverse reaction rate (ADR) (95% confidence interval (CI): 1.7–2.1%); besides, a high ADR was associated with increased frequency, extended injection duration and drug combination (RU, 2018). As reported by a study conducted to detect XYP injection-related side effects in 2018, relative to drug combination and drug dose, there were distinct age-related side effects (*p* < 0.05). In addition, relative to nervous/respiratory/digestive system, skin accounted for the major organ related to side effects (*p* < 0.05), highlighting the need of strengthening reasonable application of XYP injection (Li, 2018). Nonetheless, many side effects may return to normal level after drug withdrawal. Besides, XYP injection is not recommended for pregnant women, allergic population, and <1-year-old pediatric population. Consequently, more attention should be paid to XYP injection in clinical practice.

## Limitation

Certain limitations should be noted in this work (Papp et al., 2013): studies regarding XYP plus acitretin for treating PV are lacking, and just 10 RCTs recruiting just 815 subjects were selected in the present work, which had certain effects on result generalization and stability (Korman, 2020). Many of our included studies were conducted with reference to RCTs, but specific RCTs with generation of random sequence, outcome assessment blinding and concealment of allocation were unavailable. Our selected studies had low methodological quality, which might have caused bias (Langley et al., 2005). Despite the systematic and comprehensive search of English and Chinese databases in this study, those included articles were all published in Chinese, while foreign research support was lacking. Besides, this meta-analysis had a small sample size, possibly because that XYP is not widely used to treat PV in China (May et al., 2012). According to clinical guidelines, a 75% reduction in PASI score (PASI 75) or more was set as the successful treatment criterion (Menter et al., 2008). The Consensus of Diagnosis and Treatment of Psoriasis Vulgaris in Integrative Medicine in China set PASI 60 to be the threshold for measuring effective treatment (Association, 2009). However, none of the studies mentioned PASI 75, as a result, it is not easy for the direct comparison with results obtained in international research. PASI20-30 was used in six studies (77%) as the total effective rate criterion, and yet it fell far short of the China’s standard. Therefore, they might show higher total effective rate than the others in the results (Li et al., 2018). Besides, long-term result reporting was insufficient in this meta-analysis, therefore, it was still unknown whether those effects achieved were maintained for a long time or long-term XYP plus acitretin application was safe. According to the above mentioned limitations, we are looking forward more and more high quality clinical studies about XYP plus acitretin for PV, and many basic researches about the mechanisms of action of XYP also need to be done in the further steps.

## Conclusion

To sum up, according to this systemic review and meta-analysis, XYP plus acitretin can be an anti-PV treatment with high effectiveness and safety. Nonetheless, given the insufficient safety data and low methodological quality, more high-quality studies should be conducted in the future for proving its effectiveness and providing sound evidence for its application in clinic.

## Data Availability

The original contributions presented in the study are included in the article/Supplementary Material, further inquiries can be directed to the corresponding author.
